# Updates on Pharmacologic Management of Microvascular Angina

**DOI:** 10.1155/2022/6080258

**Published:** 2022-10-25

**Authors:** Mosayeb Soleymani, Farzad Masoudkabir, Mahsima Shabani, Ali Vasheghani-Farahani, Amir Hossein Behnoush, Amirmohammad Khalaji

**Affiliations:** ^1^Students' Scientific Research Center, Tehran University of Medical Sciences, Tehran, Iran; ^2^Cardiac Primary Prevention Research Center, Cardiovascular Diseases Research Institute, Tehran University of Medical Sciences, Tehran, Iran; ^3^Department of Cardiac Electrophysiology, Tehran Heart Center, Tehran University of Medical Sciences, Tehran, Iran; ^4^Division of Cardiology, Department of Medicine, School of Medicine, Johns Hopkins University, Baltimore, MD, USA; ^5^Non-Communicable Diseases Research Center, Endocrinology and Metabolism Population Sciences Institute, Tehran University of Medical Sciences, Tehran, Iran

## Abstract

Microvascular angina (MVA), historically called cardiac syndrome X, refers to angina with nonobstructive coronary artery disease. This female-predominant cardiovascular disorder adds considerable health-related costs due to repeated diagnostic angiography and frequent hospital admissions. Despite the high prevalence of this diagnosis in patients undergoing coronary angiography, it is still a therapeutic challenge for cardiologists. Unlike obstructive coronary artery disease, with multiple evidence-based therapies and management guidelines, little is known regarding the management of MVA. During the last decade, many therapeutic interventions have been suggested for the treatment of MVA. However, there is a lack of summarization tab and update of current knowledge about pharmacologic management of MVA, mostly due to unclear pathophysiology. In this article, we have reviewed the underlying mechanisms of MVA and the outcomes of various medications in patients with this disease. Contrary to vasospastic angina in which normal angiogram is observed as well, nitrates are not effective in the treatment of MVA. Beta-blockers and calcium channel blockers have the strongest evidence of improving the symptoms. Moreover, the use of angiotensin-converting enzyme inhibitors or angiotensin receptor blockers, statins, estrogen, and novel antianginal drugs has had promising outcomes. Investigations are still ongoing for vitamin D, omega-3, incretins, and *n*-acetyl cysteine, which have resulted in beneficial initial outcomes. We believe that the employment of the available results and results of the future large-scale trials into cardiac care guidelines would help reduce the global cost of cardiac care tremendously.

## 1. Introduction

Microvascular angina (MVA) was first described by Kemp in 1973 as an angina-like chest pain without any evidence of coronary obstruction in angiogram [1]. Although there has been controversy in the definition of MVA, recently in 2018, The Coronary Vasomotion Disorders International Study Group published standardized criteria for diagnosis of MVA as presence of symptoms of myocardial ischemia accompanied with evidence of ischemia on electrocardiogram (ECG) or on cardiac imaging along with the absence of obstructive coronary artery disease (CAD) and evidence of impaired coronary microcirculation [2]. Advancements in diagnostic imaging have caused a rise in the prevalence of MVA in recent years, with 50% of about 400,000 suspected CAD patients reported having normal angiography with no established CAD or nonobstructive CAD in a study across the United States [3, 4]. In the recent CE-MARC2 study, 68% of patients with angina and nonobstructive coronary angiogram had abnormal microvascular function [5]. Although, the true prevalence of MVA is not known and is difficult to establish but above data does suggest a high prevalence and high burden on health care. MVA is a female-predominant disorder with women comprising about 70% of patients MVA were diagnosed in 43.1% of women and only 14% of the men with typical angina undergoing coronary angiography [6]. In a study conducted in the United States, Hispanics contributed 21%, and non-White races accounted for 38% of MVA patients [7].

Although earlier MVA was considered a benign condition, the Women's Ischemia Syndrome Evaluation (WISE) study reported the 5-year risk of adverse cardiovascular events 3 to 6 times higher in women with MVA compared to normal asymptomatic women [8]. Moreover, MVA has adverse effects on patients' quality of life due to recurrent chest pain and further imposes increased lifetime cost of healthcare mostly due to frequent hospitalizations and diagnostic procedures [9, 10].

In the last decade, despite advances in our understanding of MVA, it still remains a therapeutic challenge for physicians. In this review, we primarily focus on the pharmacologic management of MVA.

## 2. Pathophysiology

Various anatomic and functional pathophysiologic mechanisms involving coronary microcirculation are proposed to lead to MVA. Of note, MVA must not be confused with vasospastic angina which classically is a disorder of major epicardial coronary arteries.

Impaired vasodilation response, either via endothelium-dependent or endothelium-independent mechanisms, is frequently implied in the pathogenesis of MVA [11]. A vasodilator-vasoconstrictor imbalance depicted as a reduced nitric oxide (NO) release as well as high plasma levels of endothelin-1 may also be a contributing factor for endothelial dysfunction in MVA patients [12–15]. In a recent study on patients with MVA and patients with vasospastic angina, resistant arteries of patients with MVA showed decreased response to acetylcholine- (ACh-) mediated vasodilation and increased response to endothelin- and thromboxane-mediated contractions. The microvascular vasoactive responses in both MVA and vasospastic angina patients tended toward an augmented vasoconstriction [16]. ACh provocation test in patients with angina and no obstructive epicardial CAD demonstrated a microvascular spasm in almost half of them, bringing ischemic ECG patterns [17]. Similarly, a European study conducted on patients with angina and normal angiogram revealed a female-male odds ratio of 4.2 in patients with MVA, suggesting that the female predominance is due to the higher sensitivity of women to develop vasomotor dysfunction at lower ACh levels [18]. Moreover, endothelium-independent microvascular dysfunction in patients with angina without obstructive coronary arteries was suggested to be associated with multiple proinflammatory and coagulation factors including von Willebrand factor and Galectin-4 [19].

Endothelial dysfunction in patients with MVA seems to be multifactorial, and it is conceivable that risk factors like hypertension, hypercholesterolemia, smoking, diabetes mellitus, and insulin resistance can contribute to its development [20]. Cosín-Sales et al. demonstrated that high plasma C-reactive protein was associated with endothelial impairment, which indicates the potential role of inflammation in the pathogenesis of MVA [21]. This inflammatory response can decrease NO bioavailability and as a result microvascular dysfunction [22]. The high prevalence of MVA in menopausal women has also suggested the pathogenic effect of estrogen deficiency in the development of endothelial dysfunction [23].

In addition to functional abnormalities, some studies support the structural abnormalities of coronary microvasculature as a mechanism for MVA [24, 25]. In a histologic study conducted by Mosseri et al. on MVA patients, myointimal proliferation, hypertrophy of media, and fibromuscular hyperplasia have been reported as possible causes of MVA development [25].

**Figure 1:**
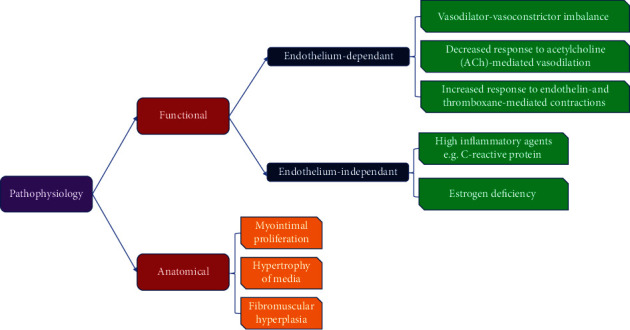
Summary of pathophysiology behind microvascular angina.

A number of studies have related the signs and symptoms of MVA patients to neurologic abnormalities including cardiac adrenergic hyperactivity, cardiac neurasthenia, and low threshold of chest pain [26, 27]. Increased activity of membrane sodium-hydrogen exchanger [28], reduced number of epithelial progenitor cells [29], and psychological morbidities [30] are among other pathophysiologic mechanisms proposed for MVA.  Figure 1 summarizes the pathophysiologic mechanisms behind MVA.

## 3. Pharmacologic Management of MVA

The majority of patients with MVA do have some extent of concomitant coronary atherosclerosis as seen in studies using the intravascular ultrasound [31]. Thus, aggressive risk factor modification and guideline-based management of hypertension, hyperlipidemia, diabetes, lifestyle changes, smoking, and obesity are indicated in most patients.

The main goal of pharmacologic interventions in patients with MVA is to control symptoms and improve patients' quality of life. Nitrates, statins, calcium channel blockers (CCBs), angiotensin-converting enzyme inhibitors (ACEI), and beta-blockers are the most commonly administered drugs for MVA patients. However, at least in part due to heterogeneous mechanisms of MVA pathophysiology, the mentioned medications have modest efficacies. Hence, the management of MVA remains a significant challenge. In the following section, we will review the mechanisms and outcomes of treatment with conventional and novel drugs in MVA patients.

### 3.1. Beta-Blockers

Blocking beta-adrenergic receptors improves myocardial ischemia by lowering heart rate, myocardial contractility, blood pressure, and consequently myocardial oxygen consumption. Additionally, beta-blockers enhance coronary blood flow (CBF) by diastolic time prolongation [32, 33]. Beta-blockers have been shown to be effective in relieving angina in about 75% of patients with MVA. In comparison to the placebo, propranolol significantly reduced the 24-h frequency of ischemic episodes (0.7 ± 0.6 vs. 3.9 ± 1.8, respectively, *p* < 0.0005) [34]. Atenolol also has been shown to reduce the number of ischemic episodes (0.44 ± 0.55 vs. 4.8 ± 4, *p* < 0.01) and improve symptoms, diastolic function, and exercise tolerance in MVA patients [35, 36]. Beta-blockers have been shown to have more favorable effects than CCBs in angina with a normal coronary angiogram [34, 36]. In comparison to the placebo, a significant decrease in the average number of daily ischemic episodes has been recorded during propranolol therapy (0.7 ± 0.6 vs. 3.9 ± 1.8, *p* < 0.0005); however, no significant association was found in the verapamil group (3.4 ± 1.7 vs. 3.9 ± 1.8) [34].

Unlike earlier generations of beta-blockers, third-generation drugs such as nebivolol and carvedilol have more endothelium-dependent vasodilatory effects. Substantial increases in the serum levels of endothelial function markers including plasma NO, L-arginine, and L-arginine/asymmetric dimethylarginine ratio have been reported in the administration of nebivolol compared to metoprolol [37]. In addition, a study by Togni et al. revealed that intracoronary nebivolol increased the coronary flow reserve (CFR) [38]. In a randomized placebo-controlled trial on MVA patients, Kaski et al. have shown that two-thirds of patients who had received carvedilol did not have angina at peak exercise (*p* < 0.01), in one-third ST segment shift was less than 1 mm during exercise tolerance test (*p* < 0.05), and these proportions were significantly higher in carvedilol group than placebo group [39]. To explain the mechanisms of these significant effects of third-generation beta-blockers on endothelium, several theories have been proposed including adenosine triphosphate (ATP) efflux which stimulates P2Y-purinoceptor-mediated NO release [40], overexpression of inducible NO synthase by beta_3_-adrenergic receptor activation [41], and decrease in reactive oxygen species (ROS) production in endothelial cells [42, 43]. Overall, it seems that beta-blockers can be the first line of treatment for MVA patients [34] and, if required, they could be used in combination with CCBs.

### 3.2. Calcium Channel Blockers

CCBs reduce myocardial oxygen demand through their negative inotropic (nondihydropyridine) and vasodilatory effects and subsequent decrease in heart afterload [44]. Molecular-based studies have indicated that CCBs are able to protect endothelium against free radical injuries [45, 46], and amlodipine as a dihydropyridine CCB has been shown to increase nitrate production [47]. In addition, effect of CCBs in reducing microvascular tone and relieving spasm [48] raises CCBs as a potential treatment for patients with MVA.

A meta-analysis compared the effects of four CCBs in vasospastic angina patients which suggested that benidipine outperformed amlodipine, nifedipine, and diltiazem in terms of attack suppression [49]. Different clinical trials have shown discordant results about the efficacy of CCBs in patients with MVA. Cannon et al. compared the efficacy of nifedipine and verapamil versus placebo in patients with angina pectoris along with angiographically normal coronary arteries and reported fewer episodes of angina, longer exercise duration, and fewer nitroglycerin consumption in CCB users [50]. While many studies support CCBs' effects on controlling angina in MVA patients [50–52], there are some studies showing conflicting results. Amlodipine showed no benefit in reducing chest pain episodes in a trial by Lanza et al. [36]. Although intravenous administration of diltiazem failed to increase CFR in patients with MVA [53], sustained-release capsules of diltiazem have been reported to significantly improve chest pain, treadmill exercise test, and CBF [52]. It has been suggested that long-acting L-type CCB is more favorable than short-acting ones for the coronary microcirculation [54]. Moreover, combination therapy of diltiazem with statins (fluvastatin) has been shown to improve coronary flow and prolong time to 1 mm ST segment depression in patients with MVA, to a higher extent than CCB or statins alone [55].

### 3.3. Statins

Statins beyond their lipid-lowering effect by inhibiting 3-hydroxy-3-methylglutaryl coenzyme A (HMG-CoA) reductase have noticeable effects on improving endothelial function through increasing NO bioavailability, antioxidant properties, and suppressing inflammatory responses [56].

A large number of clinical trials have demonstrated statins' effect on improving endothelial dysfunction [57–60]. A systematic review in 2011 also showed significant improvement in both peripheral and coronary endothelial function after statin therapy, highlighting its potential use in MVA [61]. Moreover, there are several studies that have evaluated the effect of statins on MVA patients. In a randomized placebo-controlled trial on 40 known cases of MVA, simvastatin 20 mg/day increased the relative brachial artery flow-mediated dilation by 52% [62]. In another randomized controlled trial in patients with MVA, 3 months prescription of pravastatin (40mg/day) significantly improved the exercise-induced ischemia and flow-mediated dilatation [63]. A combination of statins and CCBs has been tested on 68 MVA patients divided into three groups including fluvastatin (40 mg/day), diltiazem (90 mg/day), and a combination of fluvastatin (40 mg/day) and diltiazem (90 mg/day). At the end of 90 days, improvement in CFR (23.2%, 12.4%, and 29.1% respectively) was higher in patients who received combination therapy [55].

Taking all these lines of evidence together, statins should be regarded as a major drug class in the management of patients with MVA, particularly in combination with CCBs.

### 3.4. ACEI/ARBs

Angiotensin II is a vasoconstrictor agent which increases superoxide production by its effect on nicotinamide adenine dinucleotide phosphate -NADPH- and nicotinamide adenine dinucleotide –NADH [64, 65]. ACEIs or angiotensin receptor blockers (ARBs) through their effect on superoxide dismutase activation, thereby decreasing ROS, improve microvascular dysfunction [66, 67]. In addition, these drugs stimulate NO production by lowering the bradykinin degradation [68].

In several clinical trials, beneficial effects of an ACEI on forearm blood flow, CBF, and flow-mediated dilation have been demonstrated both in patients with CAD and in those with normal epicardial coronary arteries in the angiography [68–71]. In a double-blinded, randomized, and placebo-controlled based study, Chen et al. have found that the long-term treatment with enalapril, an ACEI, improves exercise performance and CFR in patients with MVA [71]. In the WISE trial, 16-week treatment with quinapril significantly improved CFR and angina symptoms [72]. In addition, cilazapril showed to be effective in increasing CFR in hypertensive patients in a study [73]. This effect was also observed in diabetic patients in whom coronary flow velocity increased after treatment with temocapril [74]. Although a few studies support ARBs' positive effect on microvascular dysfunction, some pieces of evidence showed no significant improvement in MVA patients [67, 75]. A randomized double-blind controlled trial examined the effect of 3-week irbesartan (150 mg daily) treatment in MVA patients which resulted in a trivial reduction in ST segment depression episodes and no significant change in treadmill exercise test [75]. It should be noted that adding eplerenone as a mineralocorticoid inhibitor to the ACEI or ARB could not reduce angina episodes and CFR in the women population [76].

To evaluate the priority of ACEI, beta-blockers, CCBs, and diuretics in MVA treatment, Higashi et al. conducted a clinical trial. In this study, the forearm blood flow response to reactive hyperemia, an index of endothelium-dependent vasodilation, was substantially higher in patients treated with ACEI than in individuals treated with either CCBs, beta-blockers, diuretic agents, or nothing [70].

Accordingly, it seems that ACEIs are highly potent medications in patients with angina and normal epicardial coronary artery angiogram, particularly in the presence of arterial hypertension. Thus overall, the evidence favors the role of ACEI in the management of MVA.

### 3.5. Antiplatelet Agents

In a study using intravascular ultrasound, it was found that there is a high prevalence of atherosclerosis in patients with chest pain and without obstructive CAD [31]. This suggests the beneficial use of thromboxane A2 (TXA2) inhibitors in MVA. These include low-dose aspirin and P2Y12 platelet inhibitors which can prevent vasoconstriction, platelet aggregation, and vascular injury. However, its combination with ACEI and statins tends to be more effective and is being trialed in Women's Ischemia Treatment Reduces Events in Nonobstructive CAD ((WARRIOR)-NCT03417388) [77].

## 4. Nitric Oxide Modulators

### 4.1. Nitrates

Direct relaxant effect of nitrates on vascular smooth muscles results from activation of the guanylyl cyclase signaling pathway by nitrate-released NO. Vasodilation induced by nitrates lowers cardiac preload and afterload and consequently reduces myocardial oxygen consumption [78, 79]. In addition, the vasodilatory effect of nitrates on coronary arteries increases myocardial blood supply, although the effect has been shown to be limited in the coronary microvasculature [80]. Hence, nitrates compose the main group for the medical management of patients with obstructive CAD. However, many studies have indicated the limited efficacy of nitrates in relieving angina in MVA patients [81, 82]. In addition, MVA patients did not experience proper symptom relief after administrating the sublingual nitroglycerin [2]. In a study conducted by Kaski et al., only 42% of patients with angina and normal epicardial coronary angiograms responded to the sublingual nitrate [83]. Russo et al. found out that the prescription of short-acting nitrates results in no improvement in exercise stress tests in MVA patients [82]. Even recent research has indicated that nitrates not only may not improve ischemia but also may worsen the ischemia through increasing endothelial dysfunction [84–86]. In a comparative study of atenolol, amlodipine, and isosorbide-mononitrate, the frequency of chest pain episodes during a 4-week follow-up of MVA patients was evaluated. Although atenolol significantly reduced the anginal episodes compared to placebo, nitrates and amlodipine demonstrated no significant difference [36]. Altogether, despite a high tendency to prescribe nitrates in the management of MVA, it seems that they have no significant benefit and cannot be recommended for the treatment of MVA [87].

### 4.2. L-Arginine

Asymmetric dimethylarginine (ADMA) and symmetric dimethylarginine (SDMA) are NO synthase inhibitors that have been shown to be higher in MVA patients who develop adverse events [68, 88, 89]. L-arginine as a precursor of NO induces coronary vasodilation and counteracts ADMA. It is suggested that alterations in arginine/NO metabolic profile in addition to a rise in oxidative stress are observed in MVA patients in a similar trend to CAD cases [90]. Clinical trials have shown heterogeneous results about the efficacy of L-arginine in patients with MVA. Although multiple studies have reported the effect of L-arginine on CBF and endothelial function improvement, there is limited knowledge about the favorable effect of L-arginine on exercise stress test and angina pectoris in MVA patients [91–93]. In a small study, Palloshi et al. reported the beneficial effect of L-arginine on exercise test and angina pectoris in hypertensive patients with angina and normal coronary angiograms [92]. Moreover, a trial conducted by Lerman et al. found that L-arginine supplementation for 6 months can have beneficial effects on endothelial function with improvement in symptoms and reduced endothelin concentrations [94]. Despite promising effects on endothelial function, there is currently insufficient evidence to support the use of L-arginine in the management of MVA.

### 4.3. Sildenafil

NO promotes muscle relaxation via the cyclic guanosine monophosphate (cGMP)-dependent mechanism [95]. Sildenafil as a phosphodiesterase type 5 (PDE-5) inhibitor prolongs NO bioavailability by blocking the nitric oxide degeneration [96].

The effect of sildenafil on improving endothelial dysfunction has been demonstrated frequently [97–100]. However, there are limited studies evaluating the effect of PDE-5 inhibitors on MVA. Denardo et al. noticed an acute improvement of CFR after PDE-5 inhibition by 100 mg oral sildenafil in MVA patients [101]. However, the long-term effect of sildenafil on CFR remains unclear. It seems that sildenafil can be a potential novel treatment for MVA, but more clinical trials are required to confirm the hypothesis. The inefficacy of nitrates compared to promising effects of PDE-5 inhibitor in MVA stems from the lack of nitrate-induced endothelial dysfunction, the transmural increase in coronary flow, and the less likelihood of developing coronary steal syndrome in sildenafil use in comparison with nitrates use [100]. In addition, sildenafil works specifically downstream of the endogenous NO pathway on the selective cGMP-induced vasodilation in affected regions and prevents unfavorable systemic effects of exogenous nitrate use [102].

### 4.4. Cilostazol

Cilostazol as a phosphodiesterase type 3 (PDE-3) inhibitor increases intracellular cyclic adenosine monophosphate with anti-inflammatory, antiplatelet, and vasodilatory effects [54]. The effects of cilostazol addition to CCBs and nitrates on vasospastic angina have been shown to be significant [103]. In addition, vasospastic angina refractory to amlodipine cases showed reduced angina frequency and intensity after administration of cilostazol [104].

### 4.5. Tetrahydrobiopterin (BH4)

Tetrahydrobiopterin is an essential cofactor of aromatic amino acid hydroxylases used in the biosynthesis of several neurotransmitters such as serotonin and catecholamines; in addition, it plays a critical role in nitric oxide production as a cofactor [105, 106].

Many studies have noted that BH4 causes significant improvement in the NO-mediated endothelial function [107–109]. Higashi et al. have indicated that BH4-ACh coinfusion acutely increases ACh-mediated vasodilation in 37 healthy subjects [110]. In addition, long-term administration of BH4 has been shown to restore the NO-mediated vasodilation [111]. Given BH4's favorable effect on endothelial function, it seems to be a promising therapy for MVA; however, its clinical efficacy remains to be determined.

### 4.6. Alpha-Blockers

Alpha-blockers have been proposed for MVA treatment due to their sympatholytic capacity which causes a reduction in the microvascular tone [112]. However, clinical trials have shown disappointing results.

Sixteen patients with MVA underwent a double-blind, placebo-controlled, crossover clinical trial for 10 weeks. Lastly, no difference in exercise duration, time to angina pectoris, and exercise time to 0.1 mV ST-segment depression has been noted between doxazosin-treated patients and placebo group [113]. In another study evaluating prazosin's effect on MVA patients, similar results have been reported [114].

In addition to the low efficacy of alpha-blockers, another limitation of their recommendation in MVA is tolerance which occurs in frequent administration [115].

## 5. Hormonal Drugs

### 5.1. Estrogen

The high prevalence of MVA in postmenopausal women implies that hormonal deficiency may be a critical factor in the development of endothelial dysfunction. Estrogen, by its property for accelerating reendothelialization and inhibiting endothelial cell apoptosis, certifies endothelial integrity [116, 117]. Furthermore, estrogen has anti-inflammatory and antioxidative properties and induces NO synthesis in human endothelial cells through a nongenomic estrogen receptor signaling [118–120].

Reduced number of chest pain episodes was observed in 17-beta-estradiol administration for postmenopausal women with cardiac syndrome X compared to placebo (3.7 episodes/10 days vs. 7.3 episodes/10 days, respectively) in a double-blind placebo-controlled study [121]. In another study, increased time to angina, time to 1 mm ST depression, total exercise time, and working capacity has been reported during 17-beta-estradiol treatment in patients with angina and no obstructive coronary artery lesions [122]. Finally, a trial was conducted on low-dose hormone therapy in postmenopausal women with no obstructive CAD. It concluded that low-dose hormone therapy could improve chest pain symptoms in addition to menopausal symptoms and increase the quality of life, despite not having an effect on ischemia or endothelial function [123].

Thus, estrogens are effective drugs in alleviating MVA symptoms; however, some pieces of evidence contradict with long-term prescription of estrogen because of safety concerns and reduction of benefits in long-term administration [119, 124].

### 5.2. Vitamin D

The inverse association of vitamin D and the renin-angiotensin-aldosterone system leads to increased vascular inflammation and endothelial dysfunction in vitamin D deficiency [125]. Increased parathormone level in response to insufficient serum vitamin D causes insulin resistance, which is a risk factor for endothelial dysfunction [126].

Studies have found significantly lower serum vitamin D levels in patients with MVA in comparison with the control group [127, 128]. Consistently, vitamin D replacement therapy in patients with MVA and low serum vitamin D3 has shown a significant improvement in the frequency of angina episodes (*p* = 0.003), exercise duration, maximal work capacity (*p* < 0.001), and maximal ST-segment depression (*p* = 0.001) [129].

Accordingly, recent studies suggest vitamin D as a novel and efficient medication for the treatment of MVA; however, more clinical trials are necessary to confirm the efficacy and safety of vitamin D supplementation therapy.

## 6. Novel Antianginal Drugs

### 6.1. Ranolazine

Increased inward late Na^+^ current which is observed in myocardial ischemia disturbs ion homeostasis through elevation of intracellular Na^+^ concentration with subsequent elevation of intracellular Ca^2+^. Ranolazine by inhibiting late sodium current in cardiomyocytes plays a significant role in relieving symptoms of myocardial ischemia [130]. A number of studies also noted ranolazine properties for endothelial function improvement [131].

Although favorable effects of ranolazine in the management of obstructive CAD were initially hopeful [132], there is a limited number of clinical trials to assess the effect of this drug in MVA patients. A randomized, double-blind, placebo-controlled trial investigating the effect of 4-week ranolazine therapy in 20 women with MVA showed physical functioning, angina stability, and quality of life improvement in the ranolazine-treated group, especially in those with CFR ≤ 3.0 [133]. Villano et al. also found ranolazine has a significant therapeutic effect on patients with nonobstructive CAD in combination with usual antiischemic therapies [134]. However, a more robust study on 128 patients with coronary microvascular dysfunction revealed no positive effect of 2-week ranolazine therapy on reported symptoms, myocardial perfusion reserve index, or diastolic filling rate and time [135]. This result changed in terms of significance after categorizing by baseline CFR. Rambarat et al. concluded that patients with CFR < 2.5 showed better myocardial perfusion and improvement in angina episodes after administration of ranolazine [136].

### 6.2. Ivabradine

Funny channels are highly expressed in sinoatrial (SA) node myocytes control *I*_*f*_ current, an important ionic current in charge of pacemaker activity of SA node. Ivabradine as a selective *I*_*f*_ current blocker lowers heart rate and subsequently reduces myocardial oxygen demand and improves oxygen supply [137].

Even though ivabradine's therapeutic effects on obstructive CAD have been demonstrated in several studies [138], there is insufficient evidence to determine its efficacy on MVA. Improved Seattle Angina Questionnaire items and EuroQoL scale have been observed in MVA patients who have undergone ivabradine treatment in comparison with the placebo group [134]. In addition, ranolazine has shown more potential advantages than ivabradine in symptomatic relief and treatment satisfaction in Villano et al.'s study [134]. Also, another randomized controlled trial demonstrated that ivabradine has effectiveness in reducing angina symptoms; however, it could not enhance microvascular function [134]. This may highlight its subsequent bradycardia effect in improving anginal symptoms rather than the microvascular effect.

Thus, ivabradine represents a novel and effective therapeutic modalities for MVA management; however, more clinical trials are warranted to empower such conclusions.

### 6.3. Fasudil

Fasudil, HA-1077, is an inhibitor of Rho-kinase, which mediates vascular smooth muscle, endothelial, and inflammatory cell function [139]. Preclinical studies demonstrated the substantial role of fasudil in inhibiting leukocyte-endothelial cell interactions via impaired neutrophil adhesion and chemotaxis [140]. In addition, it is reported that fasudil inhibits lipopolysaccharide (LPS)-triggered apoptosis of endothelial cells in pulmonary microvasculature by blocking c-Jun N-terminal Kinase (JNK) and mitogen-activated protein kinase (MAPK) pathway [141]. Rho-kinase inhibitors also diminish neutrophil-induced increase in endothelium permeability [142].

Even though the major therapeutic use of fasudil is pulmonary arterial hypertension treatment, a few studies report beneficial effects of this drug on vasospastic, microvascular, and stable effort angina. Fasudil therapy administered intracoronary (300 *μ*g/min for 15 min) decreases pacing-induced angina symptoms, the magnitude of ST depression, and the lactate production [143]. Another study revealed that fasudil ameliorates ACh-induced myocardial infarction (*p* < 0.01) and lactate production (*p* = 0.0125) in patients with angina and normal coronary angiogram [144].

Taken together, even though there are reports of targeting Rho-kinase for the treatment of microvascular angina, further supporting evidence is mandatory.

## 7. Miscellaneous Medications

### 7.1. Trimetazidine

In low oxygen supply conditions, shifting from glucose oxidation to free fatty acid (FFA) beta-oxidation leads to greater oxygen consumption, intracellular acidosis, and ROS formation [145, 146]. Trimetazidine by inhibiting the long-chain of 3-ketoacyl coenzyme A thiolase (LC 3-KAT) and inducing pyruvate dehydrogenase activity suppresses FFA beta-oxidation and restores homeostasis between glucose oxidation and glycolysis [146, 147].

Discordant results have been reported about the efficacy of trimetazidine in the treatment of MVA. Nalbantgil et al. examined the effect of trimetazidine on 35 patients with nonobstructive CAD in a placebo-controlled, double-blind study. They have found no change in heart rate, blood pressure at rest, peak exercise, and the time of 1 mm ST segment depression by trimetazidine intake; however, prolonged total exercise time and time to 1 mm ST depression have been noted in the trimetazidine-treated group compared with placebo [148]. Also, the study by Leonova et al. concluded that adding trimetazidine to standard therapy led to improved symptoms, quality of life, and exercise tolerance via increased myocardial perfusion and endothelial function [149]. Nevertheless, Leonardo et al. have noted no significant effects for trimetazidine in patients with MVA [35].

### 7.2. Proton Pump Inhibitors

According to a hypothesis, exposure of distal esophageal mucosa to gastric acid triggers esophagocardiac reflex, leading to coronary vasoconstriction [150]. The development of CAD symptoms with normal epicardial coronary artery angiogram in these patients shows that antiacid medications may be efficient in the treatment of MVA [151, 152].

Dietrich et al. conducted a study on 72 patients with MVA with gastroesophageal reflux disease (GERD) to assess the effect of proton pump inhibitor (PPI) on relieving angina. After eight weeks of PPI therapy in doubled standard dose, statistically significant improvement has been achieved in intensity, frequency, and duration of symptoms (*p* < 0.001) [153]. Although the study has shown the therapeutic effect of PPI on MVA, to understand the exact relation between GERD and MVA and to confirm PPI efficacy in the treatment of non-obstructive CAD in patients simultaneously suffering from acid reflux disease, further assessment is required.

### 7.3. Thiazolidinediones

Thiazolidinediones, a class of antidiabetic medications used in type-2 diabetes mellitus, have been demonstrated to be efficient in improving endothelial dysfunction through modulation of oxidative processes [154, 155]. A considerable reduction in the levels of C-reactive protein, endothelin-1, and ADMA and consequently flow-mediated dilatation have been reported in patients with metabolic syndrome after 8 weeks of rosiglitazone administration [156].

Although positive effects of thiazolidinediones on endothelial dysfunction have been determined in several studies; however, their effects on patients with MVA have not been examined.

### 7.4. Metformin

Metformin as an antidiabetic agent commonly prescribed for type-2 diabetes has been shown to be beneficial in reducing myocardial infarction in diabetic patients [157]. The role of metformin on endothelial function in diabetic patients has been investigated and shown to have significant improvement in acetylcholine-stimulated flows compared with placebo [158]. A randomized double-blind placebo-controlled study by Jadhav et al. assessed the effect of metformin in nondiabetic women patients with a normal coronary angiography but positive exercise tolerance test. It was concluded that metformin could improve vascular function and decrease myocardial ischemia [159]. However, the mechanism involved and larger trials with longer follow-ups need further research.

### 7.5. SGLT Inhibitors

Sodium-glucose cotransporter (SGLT) channel 2 inhibitors as an antidiabetic medication have proven to have beneficial effects on cardiovascular outcomes including cardiovascular death and heart failure in people with or without diabetes [160–162]. An in vitro study investigated the effects of these inhibitors on endothelium and suggested that observed cardiovascular benefits of them may be due to their action on endothelium other than mentioned myocardial and renal effects [163]. Further research assessing their effect on MVA in human studies is warranted.

### 7.6. Endothelin Receptor Antagonist

Endothelin (ET-1) increases vascular tone and causes vasoconstriction, especially in coronary arteries [164, 165]. ET_A_ and ET_b_ are two receptors mediating ET-1, from which ET_A_ is involved in the coronary vasoconstriction [166, 167]. It has been shown that circulating ET-1 is increased in MVA patients which can have negative effects on vascular function and reduce CFR [15, 168].

Due to the possible effects of endothelin receptors antagonist on MVA, a randomized double-blind controlled trial investigated the effects of ET-1 receptor antagonist atrasentan on patients with multiple cardiovascular risk factors, nonobstructive CAD, and coronary endothelial dysfunction. It concluded that 6-month treatment with atrasentan could enhance coronary microvascular endothelial function [169]. The possible genetic role of ET-1 in the pathogenesis of coronary microvascular dysfunction has been investigated as well. It is reported that ET-1 dysregulation may be the cause of disease and opens up the opportunity for further research for precision medicine using the gene therapy [170].

### 7.7. Xanthine Derivatives

Adenosine, besides its role in blood flow regulation through vasodilatory effects, is the major mediator of ischemic pain perception [171, 172]. As mentioned, pain hypersensitivity is one of the pathophysiologic mechanisms proposed for the pathogenesis of MVA, which can be modulated by xanthine derivatives as adenosine receptor blockers [172]. Another illustrated mechanism for the antianginal effect of xanthine derivatives is redistribution of blood flow toward ischemic myocardial areas through constriction of nondysfunctional microvasculature induced by inhibition of the vasodilatory effect of the adenosine [173].

A double-blind crossover study has indicated beneficial effects on exercise-induced angina in MVA patients who had received oral aminophylline for three weeks. However, no difference between aminophylline-treated patients and placebo group in frequency of ST depression and peak exercise ST depression has been noted [174]. Afterward, another small study demonstrated that aminophylline infusion could lengthen the time before the occurrence of ischemia in patients with cardiac syndrome X during the treadmill exercise test, in addition to beneficial effects on exercise-induced chest pain [175]. A few clinical trials with a limited number of participants have reported improvements in exercise-induced angina and ischemic ECG changes in MVA patients who underwent xanthine derivative treatment [176–178]. These results reveal the need for further research on the management of MVA pain sensitivity via xanthine derivatives.

Allopurinol, a xanthine oxidase inhibitor, has established a positive role in myocardial function improvement in ischemic injury and endothelial dysfunction [179]. The study by Erdogan et al. demonstrated that lower serum uric acid is associated with higher CFR and better coronary microvascular function, suggesting the administration of allopurinol for MVA. A single trial on 19 patients receiving either high-dose allopurinol or placebo was conducted. Despite a reduced serum BNP level in the allopurinol group, there was no difference in maximum exercise time, coronary flow reserve, and flow-mediated vasodilatation of the brachial artery [180].

### 7.8. Potassium-Channel Opener (Nicorandil)

Hyperpolarization induced by the opening of K^+^ channels in cell membranes leads to arterioles' smooth muscle relaxation and subsequently blood flow augmentation in ischemic areas [181]. Additionally, nicorandil, a potassium-channel opener, causes vasodilation through its nitrate property [182].

Nicorandil treatment was significantly associated with longer total exercise time and prolonged time to 1 mm ST depression (*p* = 0.036 and 0.026, respectively) compared with placebo treatment [183]. It also improved coronary flow reserve in patients with angina and normal coronary arteriograms, although the study had no control group [184]. Finally, a recent meta-analysis on the effects of nicorandil on MVA patients showed its potential of improving angina symptoms, ECG, and endothelial dysfunction. However, due to low-quality evidence among analyzed studies, clinical benefits remained unclear [185].

Although larger studies with sufficient follow-up are required, potassium-channel openers appear to be highly potent antiangina drugs that can be used in the treatment of MVA.

### 7.9. Imipramine

Imipramine, a tricyclic antidepressant (TCA), has been shown to elevate the pain threshold through the inhibition of serotonin and adrenaline reuptake [186]. Imipramine analgesic effect might be helpful for MVA patients, particularly in the presence of enhanced painful perception.

In a randomized, double-blind, placebo-controlled trial, the effect of imipramine in patients with chest pain and normal coronary angiogram was tested, which resulted in a statistically significant reduction in angina episodes in imipramine-treated patients in comparison with the placebo group (1 ± 86 vs. 52 ± 25, *p* = 0.03) [187]. Cox et al. also reported significantly fewer chest pain episodes during treatment compared to placebo in patients with angina and normal epicardial coronary arteries (11 (3–22) vs. 21 (16–28)—median (interquartile range); *p* = 0.01). However, 83% of the treated group have shown imipramine side effects including dry mouth, dizziness, nausea, and constipation, and even three patients had to be withdrawn from the study [188].

Although imipramine causes a significant reduction in chest pain episodes in patients with angina and normal angiogram, especially in whom remain symptomatic despite conventional antianginal therapy, the high incidence of side effects limited its use in MVA management.

### 7.10. Omega-3

Omega-3 fatty acids as polyunsaturated fatty acids (PUFAs) have beneficial effects on vascular integrity and endothelial dysfunction by modifying inflammatory cytokine expression and inhibiting oxidative stress [189, 190].

Bozcali et al. evaluated the effect of 4-month treatment with omega-3 in MVA patients. They have reported a substantial increase in flow-mediated dilation (from 47 ± 48 to 104 ± 23%, *p* < 0.05) and nitroglycerin-mediated dilatation (from 51 ± 53 to 93 ± 35%, *p* < 0.05), and malondialdehyde, a marker for oxidative stress, significantly decreases (4.4 ± 0.86 to 3.35 ± 0.33 mmol/L, *p* = 0.012) in patients who had received omega-3. Significant improvement in MVA symptoms also has been noted in omega-3-treated group [191]. During stress echocardiography, MVA patients had fewer repetitions of ST-segment depression when pretreated with n-3 PUFAs [192].

Thus, omega-3 has shown to be a favorable treatment in patients with nonobstructive CAD. However, further assessment is warranted to qualify for omega-3 efficacy in the management of MVA.

### 7.11. Incretin

Glucagon-like peptide-1 (GLP-1) is an incretin hormone that improves glycemic control through stimulating insulin secretion, inhibiting glucagon release and appetite suppression [193, 194]. Laboratory studies have proposed several positive cardiovascular effects for GLP-1. A 5 h incubation of human umbilical vein endothelial cell (HUVEC) cultures with liraglutide (0.1–100 *μ*g/mL) a GLP-1 receptor agonist-induced endothelial NO synthase phosphorylation and subsequently increased NO production via a 5′AMP-activated protein kinase (AMPK)-dependent pathway [195]. Moreover, reduced ROS and vascular cell adhesion molecule-1 (VCAM-1) mRNA expression have been reported in HUVEC treated with GLP-1 (0.03 and 0.3 nmol/L for 4 h) following exposure to advanced glycation end products (100 *μ*g/mL glycated bovine serum albumin) [196].

Clinical studies also demonstrated favorable effect of incretin on coronary and forearm blood flow. Basu et al. have shown enhancement in ACh (2–8 *μ*g/100 mL)-induced forearm blood flow in healthy volunteers (*n* = 10) undergoing GLP-1 infusion (1.2 pmol/kg/min), whereas GLP-1 did not affect sodium nitroprusside–regulated blood flow (0.5–2 *μ*g/100 mL) [197]. Furthermore, improved endothelial function after GLP-1 infusion (2 pmol/kg/min) has been noted in 12 fasted patients with type-2 diabetes mellitus and stable CAD [198]. In contrast, a trial in overweight participants showed no effect of intact GLP-1, protected from dipeptidyl-peptidase 4 mediated degradation on coronary microcirculation [199].

Although incretin seems to have therapeutic properties for MVA management, clinical studies are warranted to demonstrate its efficacy and safety in patients with MVA.

### 7.12. NAC

N-acetylcysteine (NAC), a thiol, is a prodrug to L-cysteine, which converts to the biologic antioxidant, and glutathione. Hence, NAC modifies NO half-life and potentiates the activity of NO by forming NO adducts [200–202].

Andrews et al. conducted a study on 16 patients undergoing cardiac catheterization (nine without obstructive lesion in epicardial coronary artery) testing the effect of NAC on ACh-mediated coronary vasodilation. They have shown 36 ± 11% (*p* = 0.02) elevation in coronary and femoral blood flow after NAC administration [203].

Taken together, NAC potentially is an effective medication to manage MVA, but more pieces of evidence are required to support its efficacy.

## 8. Recent Trials on MVA

Besides the mentioned treatments, there are some newly-introduced ones that should be considered and investigated in detail. A summary of treatment type, duration of treatment, and main findings in recently published trials on the management of MVA are available in Table 1. Mentioned trials are very insufficient to draw conclusions about the optimal drug in the treatment of MVA, and the need for new trials with larger sample sizes and investigation of different drugs is strongly recommended.

## 9. Conclusion

**Figure 2:**
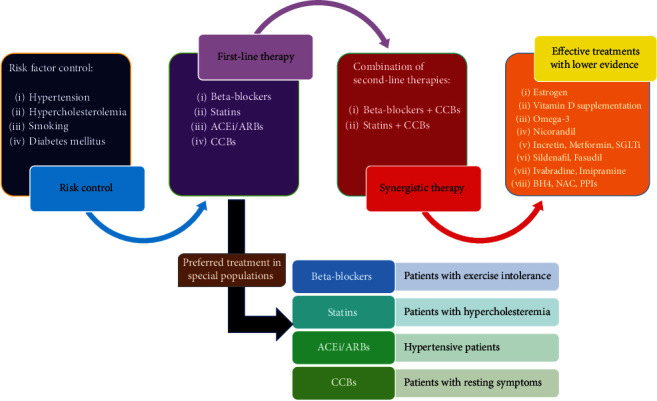
Proposed pharmacologic management algorithm for patients with microvascular angina.

Although the heterogeneity in the pathophysiology of MVA makes it difficult to control the disease in a fixed pattern, traditional anti-ischemic medications are the first line of MVA pharmacologic management. Beta-blockers, statins, ACEI/ARBs, and CCBs are the most efficient classes of drugs and should still remain the first choice of MVA treatment. If single-drug therapy is not effective, a combination of beta-blockers or statins with CCBs can be proposed (Figure 2). Also, in patients with variable thresholds of exercise-induced angina, combination therapy can be the first choice. A notable finding that differentiates the treatment of MVA from other normal-angiogram anginas like vasospastic angina is that nitrates have not been shown to work effectively in patients with MVA, yet PDE-5 inhibitors including sildenafil had therapeutic efficacy in this group. In the case of insufficient control of symptoms with the first-line medications, other available drugs including nicorandil, estrogen, imipramine, PPIs, and fasudil adjusted to patients' characteristics, can be considered (Figure 2). Novel antianginal drugs such as ranolazine or ivabradine and xanthine derivatives have been represented as the last resources for pharmacologic treatment in patients with refractory MVA [204, 205] (Table 2). Some medications are anticipated to arise as highly effective drugs in controlling MVA, including omega-3 fatty acids and vitamin D. Besides, some medications like incretin have mechanistic rationale to improve MVA, but there is no clinical study to test their efficacy in the treatment of MVA. Proposed steps of treatment for MVA are illustrated in Figure 2. In addition, the classification of drugs for the management of MVA and their proposed mechanisms are available in Table 2.

To sum up, MVA seems to have received insufficient attention from clinicians and researchers up to now. However, according to our investigations, beta-blockers, statins, ACEI/ARBs, and CCBs in addition to risk factor control may be helpful in the management of MVA. The only way to assess the efficacy and safety of suggested treatments for MVA is through large clinical trials. We hope that future trials on the treatment of this tricky yet burdensome cardiovascular issue will yield definite therapeutic strategies.

## Figures and Tables

**Table 1:** Recently-published articles on management of MVA.

Study	Design	Treatment	Duration	Results	Ref
Henry et al. [206]	Pilot clinical trial	Autologous CD34+ stem cell therapy	180 days	(i) Improved coronary flow reserve (2.08 ± 0.32 changed to 2.68 ± 0.79; *p* < 0.005) (ii) Decreased angina frequency (*p* < 0.004) (iii) Improved Canadian cardiovascular society class (*p* < 0.001) (iv) Improved quality of life (*p* ≤ 0.04)	[206]

Zhang et al. [55]	RCT	Group 1: fluvastatin (statin; 40 mg daily) Group 2: diltiazem (CCB; 90 mg daily) Group 3: statin and CCB	90 days	(i) Improved coronary flow reserve (23.2%, 12.4%, and 29.1% in groups 1 to 3, respectively; *p* < 0.05) (ii) Increased time to 1 mm ST segment depression (241 ± 97 to 410 ± 140 s, *p* < 0.05 in group 1; 258 ± 91 to 392 ± 124 s, *p* < 0.05 in group 2, and 250 ± 104 to 446 ± 164 s, *p* < 0.05 in group 3)	[55]

Kabaklić et al. [207]	Pilot RCT	Atorvastatin (20 mg daily)	90 and 180 days	(i) Improved flow-mediated dilation (*p* < 0.001 for 90 and 180 days) (ii) No difference in reactive hyperemia index (iii) Insignificant improvement in rate-normalized augmentation index (*p* = 0.077)	[207]

Makarewicz-Wujec et al. [208]	RCT	DASH diet	12 months	(i) Insignificant reduction in RANTES (42.7 ± 21.1 to 38.1 ± 18.5, *p* = 0.134) (ii) Reduced CXCL4 (12.38 ± 4.1 to 8.36 ± 2.3, *p* < 0.001)	[208]

Ref: reference; RCT: randomized controlled trial; CCB: calcium channel blocker; DASH: dietary approaches to stop hypertension.

**Table:** (a) First-line therapy or synergistic combination therapy

Medication class	Example	Mechanism of action
Beta-blockers	(i) Propranolol (ii) Atenolol (iii) Carvedilol (iv) Nebivolol	(i) Lowering heart rate, myocardial contractility, blood pressure, and oxygen consumption (ii) Endothelium-dependent vasodilatory effects through increasing plasma NO (endothelium-dependent)

Statins	(i) Pravastatin (ii) Fluvastatin	(i) Improving endothelial function through increasing NO bioavailability (endothelium-dependent and anatomical)

ACEI/ARBs	(i) Enalapril (ii) Quinapril (iii) Irbesartan (iv) Eplerenone	(i) Vasoconstriction through increasing superoxide production by its effect on nicotinamide adenine dinucleotide phosphate -NADPH- and nicotinamide adenine dinucleotide (endothelium-dependent) (ii) Stimulating NO production by lowering bradykinin degradation

Calcium channel blockers	(i) Amlodipine (ii) Nifedipine (iii) Verapamil (iv) Diltiazem	(i) Negative inotropic and vasodilatory effects (reducing microvascular tone and relieving spasm), therefore, reducing afterload (endothelium-dependent) (ii) Protecting endothelium against free radical injuries (endothelium-independent) (iii) Increasing nitrate

Antiplatelet	(i) Aspirin	(i) Inhibition of thromboxane A2 (TXA2) (endothelium-dependent)

**Table:** (b) Effective treatments with lower level of evidence

Medication class	Example	Mechanism of action
Nitric oxide modulators	(i) L-arginine (ii) Sildenafil (iii) Cilostazol (iv) Tetrahydrobiopterin	(i) Vasodilation induced by nitrates through activation of the guanylyl cyclase signaling pathway (endothelium-dependent)

Hormonal drugs	(i) Estrogen	(i) Accelerating reendothelialization (endothelium-independent) (ii) Inhibiting endothelial cell apoptosis
	(ii) Vitamin D	(iii) Decreased vascular inflammation and improving endothelial function (endothelium-independent)

Novel antianginal	(i) Ivabradine	(i) Lowering heart rate and reducing myocardial oxygen demand
	(ii) Fasudil	(ii) Mediates vascular smooth muscle, endothelial, and inflammatory cell function

Miscellaneous	(i) PPI	(i) Inhibiting esophagocardiac reflex which leads to coronary vasoconstriction (endothelium-dependent)
	(ii) Metformin	(ii) Significant improvement in acetylcholine-stimulated flows (endothelium-dependent)
	(iii) SGLT inhibitors	(iii) Action on endothelium-not know yet (endothelium-dependent)
	(iv) Endothelin receptor antagonist	(iv) Decreasing vascular tone and causing vasodilation (endothelium-dependent)
	(v) Nicorandil	(v) Arterioles' smooth muscle relaxation (vi) Vasodilation through nitrate
	(vi) Imipramine	(vii) Elevating pain threshold
	(vii) Omega-3	(viii) Modifying inflammatory cytokine expression and inhibiting oxidative stress (endothelium-independent)
	(viii) Incretin	(ix) Endothelial nitric oxide synthase (eNOS) phosphorylation and increasing NO
	(ix) NAC	(xi) Modifying NO half-life and potentiates the activity of NO

**Table:** (c) Less effective drugs

Medication class	Example	Mechanism of action
Nitric oxide modulators	(i) Nitrates	(i) Vasodilation induced by nitrates through activation of the guanylyl cyclase signaling pathway (endothelium-dependent)

Alpha-blockers	(i) Doxazosin	(i) Sympatholytic capacity which causes a reduction in the microvascular tone (endothelium-dependent)

Novel antianginal	(i) Ranolazine	(i) Inhibiting late sodium current in cardiomyocytes (ii) Improving endothelial function (endothelium-dependent)

Miscellaneous	(i) Trimetazidine	(i) Inhibiting the long-chain of 3-ketoacyl coenzyme A thiolase
	(ii) Thiazolidinediones	(i) Improving endothelial dysfunction through modulation of oxidative processes (endothelium-independent)
	(iii) Xanthine derivatives	(i) Vasodilation (endothelium-dependent) (ii) Mediating ischemic pain perception

NO: nitric oxide; ACEI: angiotensin-converting enzyme inhibitor; ARB: angiotensin receptor blockers; NADPH: nicotinamide adenine dinucleotide phosphate; SGLT: sodium-glucose cotransporter; NAC: N-acetylcysteine.
